# A Self-Supervised Adversarial Deblurring Face Recognition Network for Edge Devices

**DOI:** 10.3390/jimaging11070241

**Published:** 2025-07-15

**Authors:** Hanwen Zhang, Myun Kim, Baitong Li, Yanping Lu

**Affiliations:** 1 Department of Industrial Design, Pukyong National University, 45, Yongso-ro, Nam-Gu, Busan 48513, Republic of Korea; zhanghanwen@pukyong.ac.kr (H.Z.); luyanping@pukyong.ac.kr (Y.L.); 2 Department of Product Design, Tianjin Sino-German University of Applied Sciences, No. 1310, Dagu South Road, Hexi District, Tianjin 300220, China; libaitong@tsguas.edu.cn

**Keywords:** generative adversarial network (GAN), facial recognition, deblurring processing, Feature pyramid, global loss function, human activity recognition

## Abstract

With the advancement of information technology, human activity recognition (HAR) has been widely applied in fields such as intelligent surveillance, health monitoring, and human–computer interaction. As a crucial component of HAR, facial recognition plays a key role, especially in vision-based activity recognition. However, current facial recognition models on the market perform poorly in handling blurry images and dynamic scenarios, limiting their effectiveness in real-world HAR applications. This study aims to construct a fast and accurate facial recognition model based on novel adversarial learning and deblurring theory to enhance its performance in human activity recognition. The model employs a generative adversarial network (GAN) as the core algorithm, optimizing its generation and recognition modules by decomposing the global loss function and incorporating a feature pyramid, thereby solving the balance challenge in GAN training. Additionally, deblurring techniques are introduced to improve the model’s ability to handle blurry and dynamic images. Experimental results show that the proposed model achieves high accuracy and recall rates across multiple facial recognition datasets, with an average recall rate of 87.40% and accuracy rates of 81.06% and 79.77% on the YTF, IMDB-WIKI, and WiderFace datasets, respectively. These findings confirm that the model effectively addresses the challenges of recognizing faces in dynamic and blurry conditions in human activity recognition, demonstrating significant application potential.

## 1. Introduction

In the information age, a large amount of personal information is transmitted through the network, and the protection of personal privacy and information security have become important social issues. As an effective means of identity verification, face recognition technology is widely used in mobile payment, smart door locks, identity verification, and other fields [1]. Moreover, facial recognition is increasingly applied in human activity recognition (HAR) systems, which are essential for applications in intelligent surveillance, health monitoring, and human–computer interaction. These systems rely heavily on the accurate identification of facial features within dynamic activity environments. While deep learning-based face recognition technology has made significant progress over the past few years, especially with enhanced processing capabilities for blurry images, it still faces a number of practical challenges. In the context of HAR, these challenges are even more pronounced, as facial recognition must adapt to diverse and dynamic human movements, making robustness in blurry and variable conditions even more crucial. Firstly, although commercial facial recognition solutions show relatively good performance in normal environment recognition, the recognition accuracy of commercial systems may be less than 70% under low-light and occlusion conditions, and a high missed detection rate often occurs in the processing of blurred images [2]. Secondly, with the development of artificial intelligence technology, deep forging technology has gradually risen. This technology is capable of generating facial-looking images or videos, or even faking facial features, which poses a huge threat to existing facial recognition systems. If a face recognition system cannot accurately distinguish between real and fake images, the safety and effectiveness of its use will be seriously questioned [3,4]. When facing the increasing challenges of deepfake technology, the countermeasures for this problem include adopting advanced deep neural networks to identify and distinguish the subtle differences between real and fake images and combining multiple feature extraction and comparison algorithms to improve the detection accuracy of the system. Meanwhile, by using the method of model fusion, the training dataset and algorithm architecture are constantly updated to adapt to new forgery methods and ensure the security and reliability of the facial recognition system. This study uses GAN as the core algorithm, integrating image pyramids and deblurring principles to construct an accurate and fast facial recognition model. There are two innovative points in this study: firstly, GAN is optimized using feature pyramids. Secondly, deblurring processing is introduced in optimizing GAN. The article structure consists of four parts. The first details related works, which lays the theoretical foundation for research through a literature review. Next is the method, which in large part constructs an adversarial learning face recognition model that integrates deblurring processing. The third part is the model performance verification, which verifies its progressiveness through experiments. Finally, the conclusion is to summarize the experimental data and research limitations.

## 2. Related Works

Currently, efficient and secure facial recognition is an important guarantee for online identity verification. Faced with various scenarios, many researchers have studied and improved facial recognition technology. Srivastava G et al. [5]. proposed a modern data-driven marketing approach based on facial recognition and neuromarketing. The uniqueness of this study lies in providing the latest review of neuromarketing and facial recognition marketing, filling a gap that has not been fully studied in these two fields. Komagal E et al. [6]. proposed a student engagement analysis method based on facial expressions. In this method, multiple faces in a classroom were detected quickly and accurately through the You Only Look Once (YOLO) detector, and a robust constrained local model integration method was adopted to provide feature location for the occluded faces themselves. The system is capable of identifying behavioral activities such as concentration, non-concentration, daydreaming, napping, playing with personal items, and talking to students behind. Chen et al. [7]. proposed a lightweight face recognition algorithm to reduce the complexity of the facial feature extraction network in response to the problem of excessive parameters and computational load in facial recognition applications. The algorithm was implemented and optimized on the Jetson Nano embedded platform, enabling the face recognition system to achieve precise and real-time deployment. The system takes 37 milliseconds to complete the entire facial detection and recognition and has good robustness against complex backgrounds and illumination changes. Zhang W et al. [8]. proposed a facial expression recognition algorithm based on an improved residual neural network for the problems of network performance degradation and feature information loss in facial expression recognition. By achieving high recognition accuracy on two public datasets, the problems of reduced network performance and insufficient feature information were effectively solved. Xiao et al. [9]. found that deep cell NNs are susceptible to adversarial patch attacks. To address the security issues of face recognition models based on deep NNs, they proposed an adversarial patch model on regularized low-dimensional data manifolds. It used facial features as adversarial perturbations. After pre-training, it exhibited better transferability compared to other similar facial recognition models and also had certain advantages in recognition accuracy.

Shang L et al. [10]. proposed a unified uncertainty modeling and face recognition (FR) framework to address data uncertainty in the face recognition process. This framework adjusted the learning intensity of clean and noisy samples to improve data perception ability, showing higher performance than other models and advantages in construction cost. Due to COVID-19, wearing masks has posed challenges for face recognition. In response, Hariri W [11]. developed a method based on occlusion removal and deep learning features, which removed occluded parts and focused on extracting features of the eyes and forehead for classification using multi-layer perceptrons. This method demonstrated a higher recognition rate and reliability compared to advanced techniques. Qiu H et al. [12]. introduced a new end-to-end deep neural network model to address the recognition of occluded facial images, extracting damaged features through deep convolution and using dynamic simulation for recovery, achieving significant success on datasets like Megaface Challenge 1 and showing promise for general FR applications. Zhang L et al. [13]. found that different regions of the human face impact recognition and proposed an attention-aware facial recognition model based on deep neural networks and reinforcement learning, which utilized attention and feature networks and achieved good results in public facial verification databases, thus confirming its feasibility. Terhörst P et al. [14]. highlighted the significant impact of facial recognition on key decisions, summarizing the effects of 47 attributes on two mainstream FR systems. They proposed an improved approach to FR technology, demonstrating its effectiveness in reducing bias.

Zhang K et al. [15]. addressed the blur problem in image restoration by proposing a comprehensive literature review on deep learning image deblurring methods, discussing common causes of image blur, baseline datasets, performance indicators, and various problem representations. They classified and reviewed convolutional neural network (CNN) methods in detail based on architecture, loss functions, and applications. To tackle the challenges of spatial and temporal feature modeling due to video blur from camera shake or target motion, they introduced a deblurring network (DBLRNet) that utilizes 3D convolution for improved video deblurring performance. They further integrated DBLRNet into a generative adversarial network (GAN) architecture, employing a combination of content loss and adversarial loss for efficient training. Experimental results demonstrated that this GAN approach achieved state-of-the-art performance on two standard benchmark datasets [16]. Their work highlighted that deep learning methods, particularly CNNs, offer new solutions for deblurring by effectively capturing spatial and temporal features, enhancing feature extraction through 3D convolution. The integration of DBLRNet into the GAN framework also showcased the potential for improved blurry image recovery, using adversarial training to optimize image quality while reducing the degradation of fine-grained features that is often inherent in traditional models. To sum up, current research mostly focuses on a single technology or method, such as convolutional neural networks (CNNs), but lacks a comparative analysis of the effectiveness of different technologies in dynamic environments to illustrate the necessity of adversarial learning. Furthermore, most studies have failed to explore the limitations faced in dealing with blurred images and occlusions, which seriously affect recognition accuracy. Therefore, in order to alleviate the problem of low recognition accuracy in the case of blurred images and occlusion, this research introduces a GAN and combines deblurring processing, aiming to enhance the recognition ability of facial features in complex scenes, improve the accuracy and robustness of face recognition, and cope with increasingly complex domain challenges.

## 3. Construction of a Facial Recognition Model Based on Improved GAN and Deblurring Processing

Informatization has shifted many information authentication scenarios in daily life from offline to online, making facial authentication more frequently used in information authentication scenarios. This study constructs a fast and efficient facial recognition model based on GAN and deblurring processing.

### 3.1. Facial Recognition Model Based on an Improved Adversarial Learning Algorithm

In the field of image deblurring, architectures like ResNet, U-Net, and multi-scale methods have been successfully utilized, each with their unique strengths and limitations. ResNet is easy to train and effectively addresses the gradient vanishing problem in deep networks. However, it struggles with restoring details in blurred images and may fail to extract local features related to blurring. U-Net is favored for image segmentation due to its symmetric encoder–decoder structure, making it proficient at detail recovery. Yet, it can experience boundary blurring and oversmoothing with highly blurred images, limiting its ability to reconstruct complex details. Multi-scale methods improve detail recognition at varying scales but often incur higher computational costs and complex model designs, which may hinder real-time processing efficiency. To address the shortcomings of these architectures, an optimized GAN framework has been proposed. This structure not only generates clearer images but also validates the authenticity of the generated samples. By optimizing deblurring and image recognition in parallel within the same network, this approach enhances both the training efficiency and recognition accuracy of the model.

**Figure 1:**
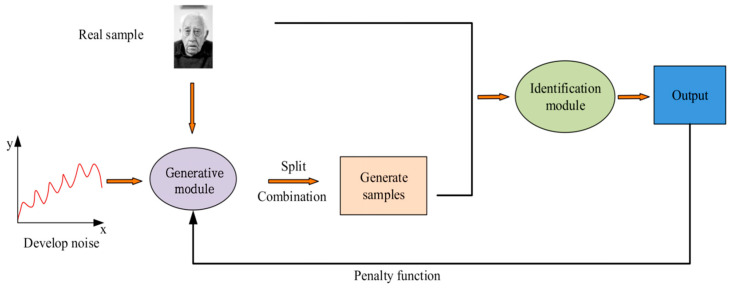
GAN model structure diagram (face image sourced from https://www.1001freedownloads.com/free-photo/nbsp-face-portrait-elder-old-wrinkles-black-and-white (accessed on 25 April 2025)).

GAN is a deep learning method derived from the two-person game, which mainly consists of two modules, discrimination and generation, both of which use deep convolutional NNs as the core method [17,18]. The generation module recombines the features extracted from the input noise samples to generate non-real samples, which are mixed with real and non-real samples as inputs to the discrimination module. The discrimination module identifies samples through multi-layer convolution and ultimately outputs the judgment results [19]. Figure 1 shows the structure of the GAN.

Figure 1 shows the basic structure and workflow of the GAN, which includes two core modules: the generator and the discriminator. In face recognition applications, the generator is responsible for generating false face images from random noise, and these images are used for comparison with real face images. The discriminator is responsible for distinguishing whether the input image is real or generated by the generator. Through this adversarial training process, the generator constantly adjusts improves its discrimination ability, enabling the model to continuously optimize its recognition performance when dealing with face images in environments such as blurry and dynamic. The loss function has a significant impact on the output results, and the global loss function of GAN is represented by Equation (1).

(1) $${min}_{G}{max}_{D}V\left(D,G\right)=E_{x\sim p_{data}(x)}\left[logD\left(x\right)\right]+E_{z\sim p_{z}(z)}[log(1-D(G(z)))]$$

In Equation (1), ${min}_{G}{max}_{D}\;$ denotes a minimax game, where the generator G and the discriminator D are optimized in an adversarial manner. $E_{x\sim p_{data}(x)}$ represents the expectation over the real data distribution $p_{data}(x)$. $E_{z\sim p_{z}(z)}$ represents the expectation over the noise distribution $p_{z}(z)$. $D\left(x\right)$ is the probability assigned by the discriminator to real samples. G(z) represents the samples generated by the generator based on noise z. $logD\left(x\right)$ and log(1−D(G(z))) denote the logarithmic probabilities of the discriminator correctly identifying real samples and incorrectly classifying generated samples, respectively. Equation (1) describes the loss function of the entire GAN model, including the gap between the real samples and the generated samples. It is used to guide the generator and discriminator on how to update parameters to optimize the quality of the generated samples and the accuracy of the discriminated samples. It is difficult to achieve the optimal parameters of Equation (1) in both the generation and discrimination modules simultaneously. Therefore, it is necessary to decompose the function and optimize its generation and discrimination modules separately. The generator module’s optimized loss function is represented by Equation (2).

(2) $$L_{G}=\frac{1}{n}\cdot \sum_{i}^{n}\log\left(1-D(G(z_{i}))\right)$$

Equation (2) focuses on the loss of the generator and represents the loss that the generator needs to minimize when generating samples. In the generator, the value is inversely proportional to the effect of generating samples. In the discriminator, the optimized loss function is represented by Equation (3).

(3) $$L_{D}=-\frac{1}{n}\cdot \sum_{i}^{n}[\log\left(D\left(x_{i}\right)\right)+\log\left(1-D(G(z_{i}))\right)]$$

Equation (3) pays more attention to the loss of the discriminant module compared to Equation (2), representing the optimization objective of the discriminator when identifying real and generated samples. In this process, the generator takes a noise vector z as input to produce fake samples G(z), while the discriminator distinguishes between real data and generated data G(z). The Nash equilibrium refers to the balance between GAN generation and recognition module training, and the biggest challenge in GAN training is finding the Nash equilibrium [20,21]. If the Nash equilibrium cannot be achieved, the model may experience long convergence time, vanishing gradients, and unstable operation. The optimized loss function has played a positive role in achieving training balance between the ride module and the recognition module, but it still requires a lot of time for parameter adjustment to achieve the optimal training [22]. However, facial recognition does not require complete recognition of all facial features of the human body, so the study refers to the cutoff method of 3D simulation training to intercept network training. This study designs an interceptor based on the perspective of facial recognition to intercept the training of GAN generation and recognition modules. Firstly, the interceptor is initialized, and the initial states of these two modules are determined, as represented by Equation (4).

(4) $$≺d-d_{1},\alpha \left(g_{0},d_{1}\right)+\beta \left(d_{1}-d_{0}\right)≻\geq 0,d_{0},d_{1}\in D$$

In Equation (4), d represents the current state of the discrimination module. $d_{1}$ refers to the state of the discrimination module after one iteration. D represents the set of discrimination records by the discrimination module. α,β are random constants in the calculation. ≺.≻ refers to the inner product. Equation (4) describes the update of the state of the discriminant module after one iteration. It improves the recognition ability of the discriminator by continuously iterating through the combination of recognition records and the current state. The interceptor adopts an iterative method for calculation. When the state $A\left(g_{i},d_{i},d_{i-1}\right)$ occurs, the current optimal training state can be obtained through Equation (5).

(5) $$\left\{\begin{array}{l} ≺g-g_{i+1},\lambda \left(g_{i+1},2d_{i}-d_{i+1}\right)+\delta \left(g_{i+1}-g_{i}\right)≻\geq 0,g\in G\\ ≺d-d_{i+1},\alpha \left(2g_{i+1}-g_{i},d_{i+1}\right)+\beta \left(d_{i+1}-d_{i}\right)≻\geq 0,d\in D \end{array}\right.$$

In Equation (5), g represents the current state of the generation module. $g_{i}$ refers to the state of the generated module after i iterations. λ,δ represent random constants in the calculation process. G refers to the collection of records generated by the generation module. $d_{i}$ represents the state of the recognition module after i iterations. Equation (5) is similar to Equation (4). This equation describes the updating of the state of the generation module after each iteration and also enhances the generation capability by combining its historical records. To simplify the calculation, G·D is denoted R, and the identity matrix is introduced, resulting in Equation (6).

(6) $$≺r-r_{i+1},D\left(r_{i+1},r_{i},r_{i-1}\right)+G^{\prime }G\left(r_{i+1},r_{i}\right)≻\geq 0,r\in R,G^{\prime }=\left(\begin{array}{l} \alpha I,0\\ 0,\beta I \end{array}\right)$$

In Equation (6), $G^{\prime }$ refers to the parameter matrix of the generation module. I is the identity matrix. r represents the overall state of GAN. Equation (6) provides a method for simplifying calculations by introducing the identity matrix, accelerating the calculation speed, reducing complexity, and facilitating the effective training of GANs, enabling effective connections and operations throughout the GAN. The termination condition of the interceptor is represented by Equation (7).

(7) $$E_{i}=\left[Max\left(||g_{i}-g_{i+1}|{|}_{\infty },||d_{i}-d_{i+1}|{|}_{\infty }\right)\right]\cdot P_{g}\left(1-P_{d}\right)$$

**Figure 2:**
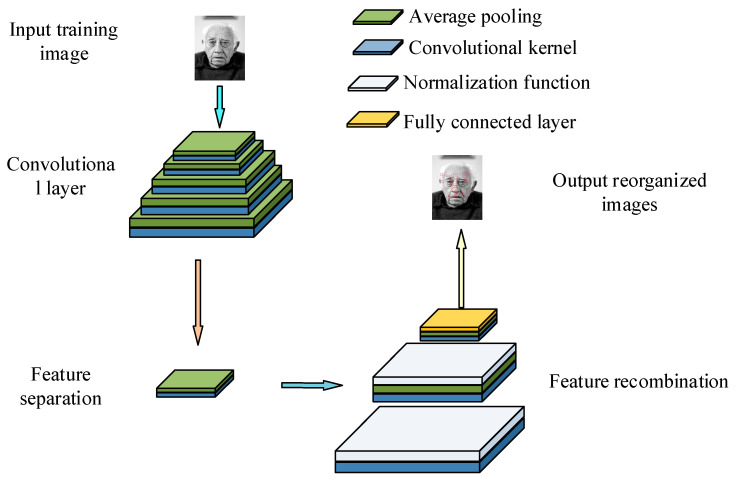
Improvement in the generation module structure diagram (face image sourced from https://www.1001freedownloads.com/free-photo/nbsp-face-portrait-elder-old-wrinkles-black-and-white (accessed on 25 April 2025)).

In Equation (7), $||.|{|}_{\infty }$ represents the ∞ paradigm. $E_{i}$ represents the cut-off point of the interceptor. $P_{g}$ represents the pass rate of the generation module. $P_{d}$ represents the recognition rate of the recognition module. Equation (7) is used to determine when the training stops, which depends on the recognition rate and pass rate of the generation module and the discrimination module. Its termination condition is that a rational stopping strategy is introduced during the training process, and the training is only stopped when the model reaches a certain performance. This dynamic adjustment mechanism avoids overtraining and helps save computing resources and improve training efficiency. When the termination conditions are met, the training classes for the generation module and recognition module will be forcibly terminated. Although the interceptor has to some extent shortened the recognition time, it still cannot meet the practical application requirements. In addition, traditional GANs often encounter several limitations during the training process, such as pattern collapse, vanishing gradients, and unstable training. These defects limit their effectiveness in complex scenarios, especially when dealing with blurred images. For instance, in a dynamic environment, GAN may fail to generate images that are clear and diverse enough, resulting in a decline in recognition performance. It is precisely because of these limitations that research has been prompted to explore improvements to GAN. By introducing an optimized loss function, feature pyramid, and deblurring techniques, the aim is to enhance the model’s ability to handle facial recognition in low-quality and dynamic scenes. Thus, this study uses image feature pyramids to improve its convolution kernel [23]. Figure 2 shows the improved generation module.

**Figure 3:**
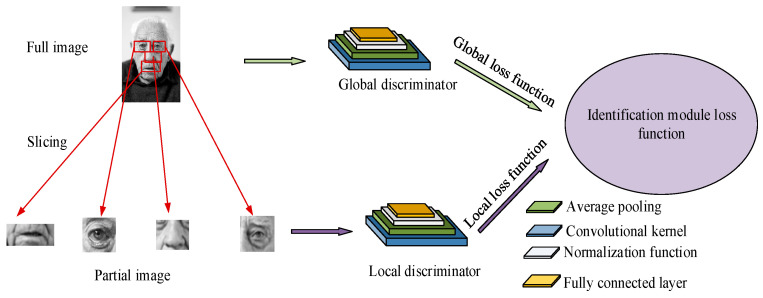
Improved identification module structure diagram (face image sourced from: https://www.1001freedownloads.com/free-photo/nbsp-face-portrait-elder-old-wrinkles-black-and-white (accessed on 25 April 2025)).

Figure 2 shows that in the improvement of the feature pyramid, this study combines the convolutional layer with the pooling layer to construct a five-layer network structure, in order to enhance the extraction ability of features at different scales. Except for the last layer, which uses 1 × 1 convolutional blocks, the remaining layers can effectively capture the fine features and important local information in the face image through the fusion of convolution and pooling operations. Furthermore, the improved feature pyramid also ensures that the model can maintain efficient feature recognition and integration capabilities under diverse inputs and fuzzy conditions by introducing a multi-layer feature fusion strategy. To further improve the overall running speed, optimization of the recognition module can also be considered [24]. Figure 3 shows the improved recognition module.

Improving the model involves two aspects; the first is improving the global discriminator by decomposing the convolution kernel from one large convolution to six small-scale convolutions, simplifying computational complexity and improving computational speed. The second is improving the local discriminator by using randomly segmented facial images as input so that the generated penalty loss function can better reflect the local features of the image. The improvement of the local discriminator is similar to that of the global discriminator, but the handling of the penalty loss function is different. The penalty loss function of the global discriminator does not require additional weights, while the penalty loss function of the local discriminator needs to be integrated after adding a set of weights. At this point, the improved GAN based on the interceptor is completed, abbreviated to I-GAN.

### 3.2. Construction of an Adversarial Learning Face Recognition Model That Integrates Deblurring Processing

Facial recognition may encounter various external factors in its application. This study finds that the face recognition model with improved adversarial learning algorithms has poor performance in processing blurred images. There are many reasons for image blurring, such as shooting jitter, lighting effects, and pixel factors, but the principle of image deblurring is generally the same [25]. Firstly, the facial edges need to be distinguished. After distinguishing the edges, the model only needs to extract features from the local area, which can further increase the recognition speed of the model. The formula for edge discrimination is represented by Equation (8).

(8) $$\theta \left(x_{v},y_{v}\right)=F\left(x_{v},y_{v}\right)\times V\left(x_{v},y_{v}\right)+\varphi \left(x_{v},y_{v}\right)$$

In Equation (8), $\theta \left(x_{v},y_{v}\right)$ means the pixel coordinates of the image edges. $\varphi \left(x_{v},y_{v}\right)$ is noise. $F\left(x_{v},y_{v}\right)$ refers to the input image. $V\left(x_{v},y_{v}\right)$ represents a fuzzy function. Equation (8) describes the process by which the model recognizes the edges in the image, helping the model focus on the key parts of the image. The blur function adjusts its parameters based on the blurriness of the input image, and its parameters are also affected by factors such as image brightness and contrast. Brightness enhancement is an important method through which to increase model deblurring. The brightness of the facial area in the image is represented by Equation (9).

(9) $$\left(x,y\right)=\sum_{\left(x,y\right)\in B}F\left(x_{i},y_{i}\right)\cdot \frac{1}{n^{2}}$$

In Equation (9), n refers to the length and width of region B. $\left(x_{i},y_{i}\right)$ represents the center point coordinates of region B. $F\left(x_{i},y_{i}\right)$ refers to the pixel set of region B. To improve the face detection rate, research is conducted on using brightness generalization technology to process the brightness of facial regions. Generalization technology can adjust facial brightness, improve the recognition effect of weak or blurry images, and have an improving effect on the output. The formula for generalization processing is represented by Equation (10).

(10) $$S\left(x_{v},y_{v}\right)=\left\{\begin{array}{c} 0,S\left(x_{v},y_{v}\right)<S_{avg}-0.5\\ 1,S\left(x_{v},y_{v}\right)>S_{avg}-0.5\\ S\left(x_{v},y_{v}\right)-S_{avg}+0.5,\;other \end{array}\right.$$

In Equation (10), $S(x_{v},y_{v})$ represents the value of the generalized pixel. $S_{avg}$ represents the average brightness of the area. The value 0.5 refers to the average quantization parameter. After generalizing the brightness, the overall brightness of the image will increase, and the gradient value of the image will also change after the brightness is increased. The gradient value is represented by Equation (11).

(11) $$\left|\phi^{\prime }\left(x,y\right)|=(\frac{\left|\phi \left(x,y\right)\right|}{1+\left(\frac{\left|B\left(x,y\right)-B_{avg}\right|}{B_{avg}}\right)}\right)$$

**Figure 4:**
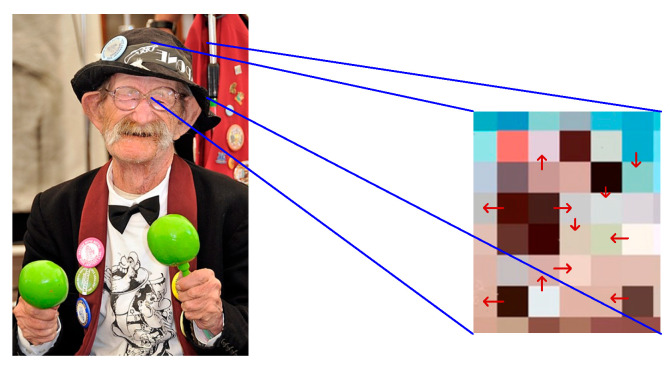
The gradient variation of image brightness (face image Source from: https://www.1001freedownloads.com/free-photo/man-face-old-person-jazz-musician-entertainer-2 (accessed on 27 April 2025)).

In Equation (11), $\left|\phi \left(x,y\right)\right|$ refers to the gradient value of the ungenerated image at $\left(x,y\right)$. $\left|\phi^{\prime }\left(x,y\right)\right|$ represents the gradient value at $\left(x,y\right)$ after generalization. $B_{avg}$ refers to the evaluated brightness of the human facial area. $B\left(x,y\right)$ means the brightness of a certain area centered on $\left(x,y\right)$. Figure 4 shows the gradient variation of image brightness. The arrows indicate the local gradient direction, which corresponds to the direction of maximum brightness change at each pixel. Note that the gradient vector may point from dark to bright or from bright to dark, depending on the local pixel intensity distribution.

Gradient calculation can reflect the trend of change in brightness from the human face to the background and can assist the model in locating and identifying the boundaries of the region. After gradient calculation, assuming that each pixel has a resolution level of q, the detailed contour of the image is expressed using Equation (12).

(12) $$T_{q}\left(x,y\right)=\left\{\begin{array}{l} \sqrt{L_{xq}^{2}\left(x,y\right)+L_{yq}^{2}\left(x,y\right)}>\xi_{j}\\ 0,Otherwise \end{array}\right.$$

In Equation (12), $L_{xq}\left(x,y\right)$ refers to horizontal pixels. $L_{yq}\left(x,y\right)$ represents vertical pixels. $\xi_{j}$ is the edge threshold. $T_{q}\left(x,y\right)$ refers to edge contour pixels. There is usually a significant difference in brightness and pixel values between the detection area and background, so it is necessary to set a threshold for the boundary between the face and other areas. When the change value is greater than this threshold, it is considered that the point is the boundary, that is, the face contour. The threshold is related to the pixel value, image size, resolution, and brightness gradient of the facial contour, and the threshold $\xi_{j}$ of the image edge is represented by Equation (13).

(13) $$\xi_{j}=\frac{2^{j-1}}{l_{j}\cdot w_{j}}\sum_{x=1}^{l}\sum_{y=1}^{w}\sqrt{L_{xq}^{2}\left(x,y\right)+L_{yq}^{2}\left(x,y\right)}$$

In Equation (13), $l_{j}$ refers to the length of the image when the resolution is j. $w_{j}$ represents the width of the image when the resolution is j. Equations (9)–(13) elaborate in detail upon how to process the face area through luminance normalization technology to improve the recognition effect, and they introduce the threshold of edge detection to enhance the perceptibility of facial edges. At this point, the probability that the model can perceive the edges of the face is represented by Equation (14).

(14) $$C_{xy}=[{\sum_{x=1}^{l}\sum_{y=1}^{w}\left|\frac{w\left(P_{j}\left(x,y\right)\right)}{w_{n}}{|}^{\kappa }\right]}^{\frac{1}{\kappa }}$$

In Equation (14), κ represents the model distortion coefficient. $w_{n}$ refers to the minimum bounding box value that this model can recognize. $P_{j\left(x,y\right)}$ is the probability that this model can detect facial edges. Equation (14) indicates the success probability of the model when detecting edges, which is related to the characteristics of the edges and reflects the sensitivity of the model. Therefore, the edge pixels of the input image should satisfy Equation (15).

(15) $$\tau_{j}\left(x,y\right)=1-\tau_{vj}\left(x,y\right)$$

**Figure 5:**
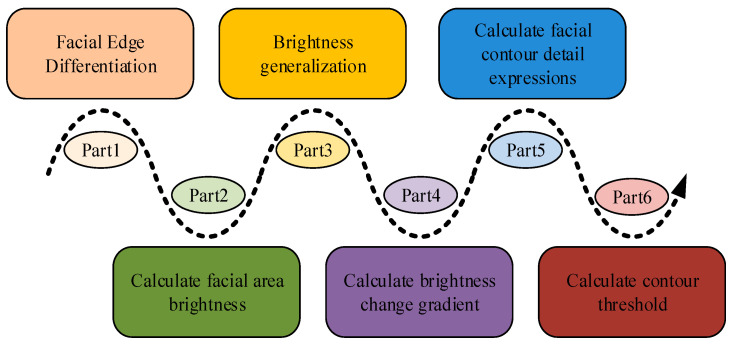
Facial contour calculation flowchart.

In Equation (15), $\tau_{j\left(x,y\right)}$ is the clarity of point $\left(x,y\right)$ at a resolution of j. $\tau_{vj}\left(x,y\right)$ represents the blurriness of point $\left(x,y\right)$ at a resolution of j. Equation (15) indicates the conditions for maintaining clarity of the image during the processing, which is closely related to the degree of blurriness, prompting the model to select an appropriate processing strategy. The above deblurring processing can further improve the recognition speed of the model and expand the application scenarios of the proposed model. Figure 5 shows the basic process of image blur processing.

After image deblurring, the model’s ability to process blurred images is further improved. And there is also optimization in model recognition speed, which can obtain accurate results more quickly, thereby improving overall processing efficiency and accuracy.

## 4. Performance Verification of Adversarial Learning Face Recognition Model with Fusion Deblurring Processing

The device used was a desktop computer with 16GB of running memory, i7-13700K CPU, and a GeForce GTX 470 graphics card. The system resource was Windows 10. The software resource was JavaSE. The datasets used for training and testing included YTF, IMDB WIKI, and WiderFace. YTF is a large-scale video dataset for facial recognition and verification, consisting primarily of facial images from YouTube videos. The dataset consists of 3,000 video clips of 341 different identities, each with a different number of facial images and their expressions, poses, and shooting angles, giving the dataset significant advantages in terms of diversity and complexity. YTF is particularly suitable for evaluating the performance of facial recognition algorithms in dynamic scenes and non-static states, while demonstrating the challenges of facial blurring, lighting changes, and occlusion in video. Imdb-wiki is a massive facial dataset, collected by both IMDB and WIKI, containing more than 500,000 facial images and covering multiple age groups and genders. The images are primarily used for age estimation and gender classification studies, and the labels included in the dataset make it suitable for training and testing multiple facial recognition and analysis models. WiderFace is a dataset dedicated to face detection that contains 32,203 images covering faces in a variety of complex scenarios, especially those taken in natural environments. There are more than 400,000 face instances labeled in this dataset, which includes a variety of facial gestures, occlusion degree, and lighting conditions, making the evaluation of algorithm performance more challenging and practical. When conducting model training and validation using datasets such as YTF, IMDB-WIKI and WiderFace, attention should be paid to potential dataset bias issues. For example, there may be insufficient representation of age, gender or race in the datasets, resulting in a decline in the model’s recognition ability among certain populations. Furthermore, the differences in image quality, acquisition conditions, and environmental diversity may also affect the robustness of the model. To address these biases, the research adopted data augmentation strategies, such as incorporating techniques like rotation, scaling, and color transformation during the training process to increase the diversity of samples. Moreover, cross-validation was conducted by fusing different datasets, thereby enhancing the generalization ability and fairness of the model.

In this research, the implementation details of the model included multiple important hyperparameter settings and the arrangement of the training cycle to ensure the optimization of its performance. Hyperparameters such as the learning rate, batch size, and the network structure of the generator and discriminator were all strictly adjusted. The learning rate was set to an initial value of 0.0002, and a dynamic adjustment strategy was adopted to adapt to different training stages. The batch size was set to 64 to balance computational efficiency and the rate of loss convergence. The training cycle was set at 50 cycles. Each cycle included real-time validation for the YTF, IMDB-WIKI and WiderFace datasets to ensure that the model can provide timely feedback and adjust the strategy during the training process to avoid overfitting. Meanwhile, data augmentation techniques, such as random cropping and rotation, were also introduced to increase the diversity of training samples, thereby further enhancing the generalization ability and robustness of the model.

**Figure 6:**
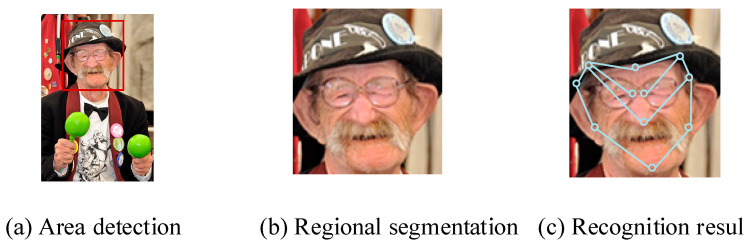
Model recognition effect display (face image sourced from https://www.1001freedownloads.com/free-photo/man-face-old-person-jazz-musician-entertainer-2 (accessed on 27 April 2025)).

To verify the region selection, segmentation, and recognition performance of this model, this study randomly selected a facial image as the input for DE-I-GAN. In Figure 6, the effectiveness of the DE-I-GAN model in facial localization and segmentation is demonstrated. By inputting specific facial images, the model can accurately identify the positions of facial feature points such as eyes, nose and mouth, indicating its strong adaptability in dynamic and complex scenes. Compared with I-GAN and GAN, DE-I-GAN significantly improves the positioning accuracy of feature points, reflecting the effectiveness of its optimization strategy for blurred and dynamic image problems.

**Figure 7:**
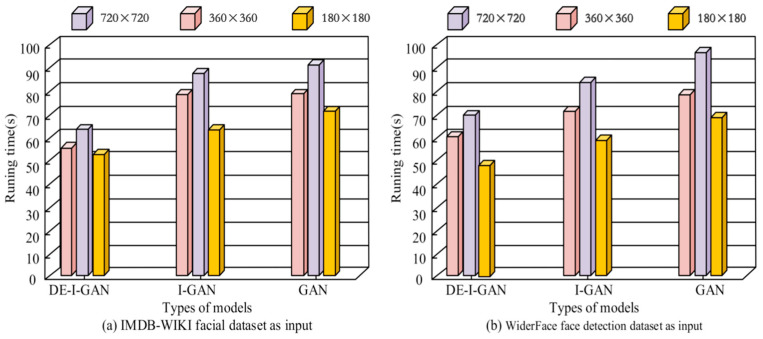
Identification time results of different models.

Facial recognition requires real-time implementation, so the model recognition speed must be very fast. To test the DE-I-GAN recognition speed, this study used I-GAN and GAN as controls. A total of 100 images with 720 × 720, 360 × 360, and 180 × 180 pixels each were selected as inputs from WiderFace and IMDB-WIKI. Figure 7 records the average output time required by DE-I-GAN, I-GAN and GAN when processing the same image size. The results show that DE-I-GAN exhibits shorter processing times than I-GAN and GAN when dealing with 720 × 720, 360 × 360, and 180 × 180-pixel images, which are 70.01 s, 60.26 s, and 47.16 s, respectively. This indicates that DE-I-GAN has made significant progress in optimizing computational efficiency and is more suitable for scenarios that require rapid response and real-time processing. In contrast, there is no significant difference in the response time between I-GAN and GAN under the same conditions, indicating the deficiency in their training strategies. Therefore, DE-I-GAN not only improves the recognition accuracy, but also increases the processing speed, making it more capable of meeting the requirements of practical applications.

**Figure 8:**
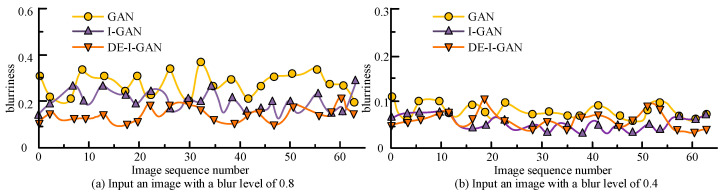
Display of deblurring effects of different models.

To verify the deblurring effect of DE-I-GAN, images with blur parameters of 0.8 and 0.4 in the YTF dataset were selected as inputs in Figure 8. Figure 8 provides a comparison of DE-I-GAN, I-GAN, and GAN in terms of deblurring. For the image with a blurring parameter of 0.8, the blurring degree of the output image of DE-I-GAN is reduced to 0.173, which is significantly better than 0.226 (I-GAN) and 0.360 (GAN). When the blurring parameter is 0.4, the blurring degree output by DE-I-GAN is also relatively low, indicating that it has a stronger deblurring ability under high blurring conditions. This result emphasizes the effectiveness of the DE-I-GAN-integrated deblurring technology, enabling the recovery of relatively clear facial feature information even when the input image quality is poor.

**Figure 9:**
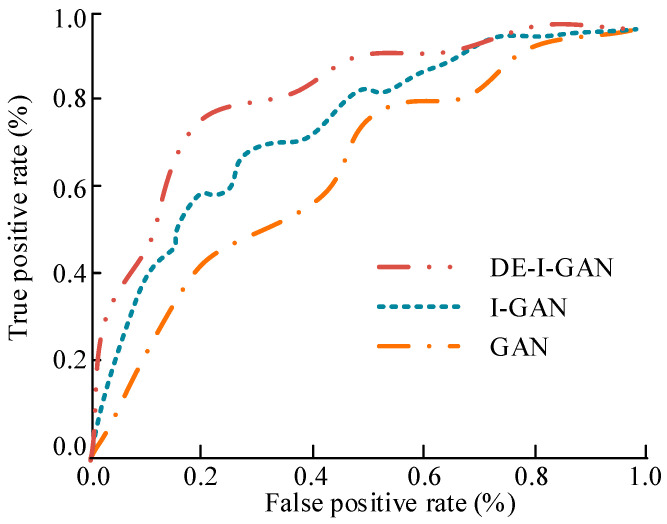
The ROC curve results of different models on the YTF dataset.

In Figure 9, the ROC curve shows the true positive case rate and false positive case rate of different models on the YTF dataset. The ROC curve of DE-I-GAN rapidly approached a 100% true positive case rate, demonstrating its high sensitivity and accuracy in recognizing facial images. In contrast, the curves of I-GAN and GAN show a relatively slow upward trend, indicating that their responses are not rapid enough when processing face images with background interference or blurriness. This further verifies the superiority demonstrated by DE-I-GAN in dynamic scenes.

**Figure 10:**
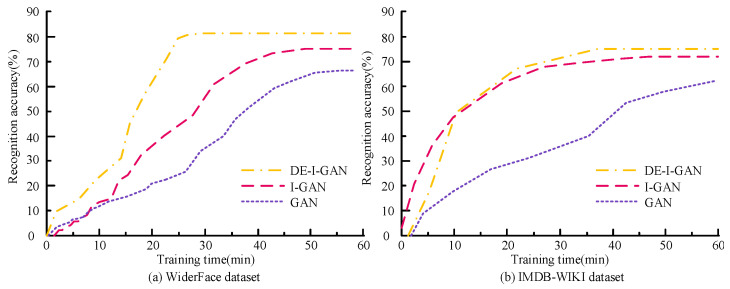
Relationship between training time and accuracy.

Figure 10 shows the varied relationship between the training time and accuracy of DE-I-GAN, I-GAN and GAN on the WiderFace and IMDB-WIKI datasets. In the WiderFace dataset, DE-I-GAN can converge rapidly, and the accuracy rate eventually reaches 81.06%, which is significantly higher than the results of the other two models. This indicates that DE-I-GAN achieves a better classification performance while maintaining a lower training time. In IMDB-WIKI, although the convergence time of DE-I-GAN is slightly longer than that of I-GAN, its accuracy rate still remains at 79.77, demonstrating the stronger stability and consistency of the model. Overall, DE-I-GAN demonstrates higher training efficiency and recognition ability, indicating that it has broader prospects in practical applications.

**Figure 11:**
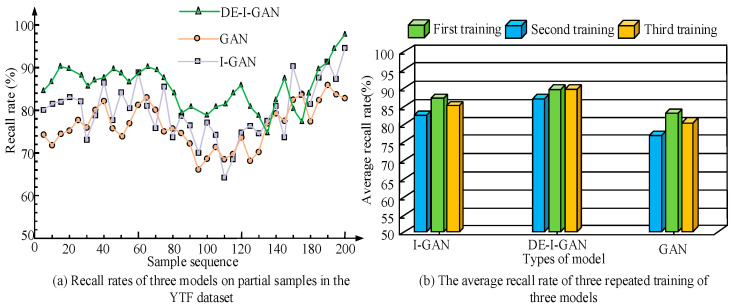
Comparison chart of recall rates of various models.

In Figure 11, the recall rates of each model on the YTF dataset are compared in detail. The results show that the average recall rate of DE-I-GAN is 87.40%, which is significantly higher than that of I-GAN and GAN. This high recall rate means that DE-I-GAN has a relatively high success rate in recognizing facial features, thereby reducing the probability of missed detection, and is suitable for application scenarios with high requirements for recognition accuracy. In contrast, the recall rates of I-GAN and GAN are relatively low, reflecting their insufficient identification ability in complex environments. This result further indicates the advantages of DE-I-GAN under dynamic and fuzzy conditions and is suitable for practical application fields such as monitoring and security.

**Figure 12:**
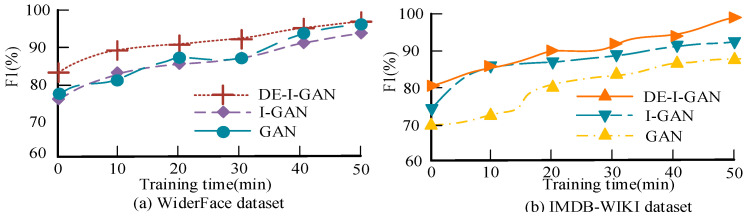
Schematic diagram comparing F1 values of various models.

Figure 12 shows the F1 value performance of DE-I-GAN, I-GAN, and GAN during the training process, which were tested on the WiderFace and IMDB-WIKI datasets, respectively. The F1 value of DE-I-GAN outperformed other control models on both datasets, reaching 96.17% on WiderFace. The increase in the F1 value shows that DE-I-GAN achieved a better balance between precision and recall, demonstrating its superior overall performance in complex facial recognition tasks.

Table 1 verifies the validity of each component of the model through ablation experiments. The basic GAN model performed inadequately in fuzzy image processing (with an average recall rate of 70.35% and an F1 value of 80.11%). The indicators improved after the introduction of the improved GAN (recall rate 74.10%, F1 value 84.20%). The performance was significantly enhanced after combining the feature pyramid (recall rate 80.55%, F1 value 90.32%). After adding the deblurring technology, the indicators continued to improve (recall rate 82.05%, F1 value 92.18%). The complete model (feature pyramid + deblurring + improved GAN) achieved the optimal effect. The average recall rate was 87.40%; the accuracy rates of IMDB-WIKI/WiderFace reached 81.06%/79.77%, respectively; and the F1 value was 96.17%, which proved that the synergy of each component was significant. Among them, the feature pyramid and deblurring technology made a key contribution to the performance improvement. To further verify the feasibility of the proposed method, a comparative analysis was conducted between the Dual Variational Generative Face (DVG-Face) model and the Adaptive Robust Face (ARFace). The experiment selected a high-resolution deblurring dataset. The sample pixels of the dataset were 720×1280, and it was used to test dynamic blur restoration. The evaluation indicators include peak signal-to-noise ratio (PSNR), structural similarity index measure (SSIM), floating-point operation cost (FLOPs), memory usage, deblurring computing cost (proportion of additional FLOPs), and interceptor analysis (convergence time). The specific results are shown in Table 2.

Table 2 presents the feasibility analysis results of different models on high-resolution deblurring datasets. Through comparison, DE-I-GAN achieved a peak signal-to-noise ratio (PSNR) of 29.7 ± 0.3 dB, significantly higher than 28.1 ± 0.4 dB of DVG-Face and 25.6 ± 0.5 dB of ARFace, indicating that DE-I-GAN has more advantages in image quality. Meanwhile, in the structural similarity index (SSIM), the 0.921 of DE-I-GAN is also superior to the other two models, further indicating its effectiveness in preserving the image structure. In terms of performance indicators, although the floating-point operation capacity (FLOPs) of DE-I-GAN is 45.2 G (which is lower than 68.7 G of DVG-Face but higher than 52.4 G of ARFace), the memory usage of 3.8 GB is also less than that of DVG-Face (5.2 GB) and ARFace (4.5 GB). It is worth noting that the proportion of additional FLOPs of DE-I-GAN is 0.15%, which is relatively low, demonstrating its efficiency in terms of the cost of deblurring computing. Finally, its convergence time of 8.2 h is also superior to the 12.5 h of DVG-Face and the 10.1 h of ARFace, highlighting the advantages of DE-I-GAN in overall training and execution efficiency. These results indicate that DE-I-GAN exhibits stronger throughput capacity and a superior image-processing effect in the dynamic blur restoration task, providing strong support for its feasibility in practical applications. To test the effect of this research method in solving the global optimal constraint, this study introduced the genetic algorithm (GA) and the particle swarm optimization algorithm (PSO) for comparison. The results are shown in Table 3.

Table 3 shows the influence of different optimization algorithms on the model effect. When the batch size of the original DE-I-GAN model was 32, the PSNR was 29.7 ± 0.3 dB, the SSIM was 0.921, and the convergence time was 8.2 h. After introducing the genetic algorithm (GA), when the batch size of the GA-DE-I-GAN model was 64, the PSNR increased to 32.5 ± 0.2 dB, the SSIM rose to 0.935, and the convergence time shortened to 6.5 h, showing a significant performance improvement. In contrast, the PSO-DE-I-GAN optimized by particle swarm optimization (PSO) has a PSNR of 31.7 ± 0.4 dB and an SSIM of 0.925 when the batch size is 56. Although it performs well, it does not achieve the effect of GA-DE-I-GAN. The results show that the introduction of GA and PSO optimization algorithms effectively improves the model performance and convergence ability.

Overall, the proposed model has high detection accuracy and stable F1 and can also achieve ideal results in the processing of blurred images. Therefore, it has a promoting effect on the development of fuzzy facial recognition and has a positive impact on the development of the facial recognition industry.

In the experimental results presented above, the research method addresses the issues of traditional GAN gradient vanishing and mode collapse by accelerating the generator’s convergence during training. This significantly enhances the quality of the generated output images and prevents the variance from diminishing due to the discriminator’s strength. After the training, the diversity and realism of the generated images are improved, and data samples that are close to reality can be generated more effectively. Aiming to alleviate the problem of poor fuzzy image processing, a defuzzy processing module is introduced, which focuses on improving the quality of fuzzy images. The test results show that on the YTF dataset, the model can significantly enhance the recognition rate of fuzzy images, and the accuracy of images with a high degree of fuzziness can be significantly improved after processing.

## 5. Conclusions

To address the low efficiency of the model in recognizing blurred images, this study proposes an improved GAN face recognition model that integrates deblurring techniques. This model has the characteristics of fast computation and recognition of GAN and enhanced recognition ability for blurred images through deblurring techniques. The model is validated herein for performance on three datasets: YTF, IMDB WIKI, and WiderFace. These experiments confirmed that the average recall rate of DE-I-GAN in YTF reached 87.40%. After convergence on IMDB WIKI and WiderFace, its accuracy reached 81.06% and 79.77%, and F1 reached 96.17% and 94.88%. Moreover, after deblurring the image with a blur degree of 0.8, the image blur degree of DE-I-GAN was only 0.713, and the blur degree was reduced by 0.627. For an image with an input blur of 0.4, the output image blur decreased by 0.311. The proposed model had a faster processing ability for images of different sizes compared to the control model, with an average processing time of 47.16 s for images of 180 × 180 pixels processed by DE-I-GAN. The average processing time for images with 360 × 360 pixels was 60.26 s. The average processing time for 720 × 720 pixel images was 70.01 s. Compared to the average processing time of I-GAN at 52.44 s, 65.89 s, and 77.64 s, DE-I-GAN had a significant advantage. In addition, this study found a flaw in the proposed model, which is that it cannot achieve global optimum. Therefore, in future studies, a variety of advanced optimization algorithms will be introduced to overcome the limitation that the current model cannot achieve global optimization, including genetic algorithms and particle swarm optimization, to explore a wider parameter space to find the best learning rate and batch size. An adaptive learning rate mechanism is introduced to speed up convergence, as well as an ensemble learning approach to improve recognition performance by combining multiple models. The research results of this model are very important for improving the accuracy and robustness of facial recognition, providing new ideas and methods for future research.

## Figures and Tables

**Table 1:** Model ablation experiment.

Experimental Configuration	Average Recall Rate (%)	IMDB-WIKI Accuracy (%)	WiderFace Accuracy (%)	F1 Value (%)
GAN	70.35	65.40	62.25	80.11
Modified GAN	74.10	72.15	68.48	84.20
Improved GAN + feature pyramid	80.55	76.89	73.15	90.32
Improved GAN + deblur processing	82.05	78.54	75.83	92.18
Feature pyramid + deblurring + improved GAN	87.40	81.06	79.77	96.17

**Table 2:** Feasibility analysis of different models.

Method	DE-I-GAN	DVG-Face	ARFace
PSNR (dB)	29.7 ± 0.3	28.1 ± 0.4	25.6 ± 0.5
SSIM	0.921	0.895	0.862
Proportion of additional FLOPs	0.15	0.22	0.3
FLOPs (G)	45.2	68.7	52.4
Memory usage (GB)	3.8	5.2	4.5
Convergence time (h)	8.2	12.5	10.1

**Table 3:** The results of overcoming the global optimal limit.

Optimization Algorithm	Optimal Batch Size	PSNR (dB)	SSIM	Convergence Time (h)
DE-I-GAN	32	29.7 ± 0.3	0.921	8.2
GA-DE-IGAN	64	32.5 ± 0.2	0.935	6.5
PSO-DE-IGAN	56	31.7 ± 0.4	0.925	7

## Data Availability

The raw data supporting the conclusions of this article will be made available by the authors on request.

## References

[1] Zhou K., Tang J. (2021). Harnessing fuzzy neural network for gear fault diagnosis with limited data labels. Int. J. Adv. Manuf. Technol..

[2] Yang L., Li Y., Yang S.X., Lu Y., Guo T., Yu K. (2022). Generative adversarial learning for intelligent trust management in 6G wireless networks. IEEE Netw..

[3] Vaidyan V.M., Tyagi A. (2023). On fuzzy inference-based supervisory control decision model with quantum artificial-intelligence electromagnetic prediction models. Int. J. Cybern. Cyber-Phys. Syst..

[4] Hasanvand M., Nooshyar M., Moharamkhani E., Selyari A. (2023). Machine learning methodology for identifying vehicles using image processing. Artif. Intell. Appl..

[5] Srivastava G., Bag S. (2024). Modern-day marketing concepts based on face recognition and neuro-marketing: A review and future research directions. Benchmarking Int. J..

[6] Komagal E., Yogameena B. (2023). PTZ-camera-based facial expression analysis using faster R-CNN for student engagement recognition. Computer Vision and Machine Intelligence Paradigms for SDGs: Select Proceedings of ICRTAC-CVMIP 2021.

[7] Chen Z., Chen J., Ding G., Huang H. (2023). A lightweight CNN-based algorithm and implementation on embedded system for real-time face recognition. Multimed. Syst..

[8] Zhang W., Zhang X., Tang Y. (2023). Facial expression recognition based on improved residual network. IET Image Process..

[9] Xiao Z., Gao X., Fu C., Dong Y., Gao W., Zhang X., Zhou J., Zhu J. Improving transferability of adversarial patches on face recognition with generative models. Proceedings of the IEEE/CVF Conference on Computer Vision and Pattern Recognition (CVPR).

[10] Shang L., Huang M., Shi W., Liu Y., Liu Y., Steven W., Sun B., Xie X., Qiao Y. Improving training and inference of face recognition models via random temperature scaling. Proceedings of the AAAI Conference on Artificial Intelligence.

[11] Hariri W. (2022). Efficient masked face recognition method during the COVID-19 pandemic. Signal Image Video Process..

[12] Qiu H., Gong D., Li Z., Liu W., Tao D. (2021). End2end occluded face recognition by masking corrupted features. IEEE Trans. Pattern Anal. Mach. Intell..

[13] Zhang L., He Z., Sun Z., Tan T. (2022). ARFace: Attention-Aware and regularization for face recognition with reinforcement learning. IEEE Trans. Biom. Behav. Identity Sci..

[14] Terhörst P., Kolf J.N., Huber M., Kirchbuchner F., Damer N., Moreno A.M., Fierrez J., Kuijper A. (2022). A comprehensive study on face recognition biases beyond demographics. IEEE Trans. Technol. Soc..

[15] Zhang K., Ren W., Luo W., Lai W.S., Stenger B., Yang M.H., Li H. (2022). Deep image deblurring: A survey. Int. J. Comput. Vis..

[16] Zhang K., Luo W., Zhong Y., Ma L., Liu W., Li H. (2019). Adversarial spatio-temporal learning for video deblurring. IEEE Trans. Image Process..

[17] Wang X. (2023). A fuzzy neural network-based automatic fault diagnosis method for permanent magnet synchronous generators. Math. Biosci. Eng..

[18] Luo N., Yu H., You Z., Li Y., Zhou T., Jiao Y., Han N., Liu C., Jiang Z., Qiao S. (2023). Fuzzy logic and neural network-based risk assessment model for import and export enterprises: A review. J. Data Sci. Intell. Syst..

[19] Kure H.I., Islam S., Ghazanfar M., Raza A., Pasha M. (2022). Asset criticality and risk prediction for an effective cybersecurity risk management of cyber-physical system. Neural Comput. Appl..

[20] Beke A., Kumbasar T. (2022). More than accuracy: A composite learning framework for interval type-2 fuzzy logic systems. IEEE Trans. Fuzzy Syst..

[21] Tseng M.L., Jeng S.Y., Lin C.W., Lim M.K. (2021). Recycled construction and demolition waste material: A cost-benefit analysis under uncertainty. Manag. Environ. Qual. Int. J..

[22] Xu P., Lan D., Yang H., Zhang S., Kim H., Shin I. (2025). Ship formation and route optimization design based on improved PSO and D-P algorithm. IEEE Access.

[23] Yu Q., Song J.-Y., Yu X.-H., Cheng K., Chen G. (2022). Solving combat-mission prediction problems with multi-instance genetic fuzzy systems. J. Supercomput..

[24] Zhang T.Y.K., Zhan J.X., Shi J.M., Xin J.M., Zheng N.N. (2023). Human-like decision-making of autonomous vehicles in dynamic traffic scenarios. IEEE/CAA J. Autom. Sin..

[25] Yang H., Xu P., Zhang S., Kim H., Shin I. (2025). Construction of an intelligent analysis system for crop health status based on drone remote sensing data and CNN. IEEE Access.

